# Cardiovascular disease in adults with osteogenesis imperfecta: clinical characteristics, care recommendations, and research priorities identified using a modified Delphi technique

**DOI:** 10.1093/jbmr/zjae197

**Published:** 2024-12-12

**Authors:** Lars Folkestad, Siddharth K Prakash, Sandesh C S Nagamani, Niels Holmark Andersen, Erin Carter, Jannie Dahl Hald, Riley J Johnson, Bente Langdahl, Eleanor M Perfetto, Cathleen Raggio, Stuart H Ralston, Robert A Sandhaus, Oliver Semler, Laura Tosi, Eric Orwoll

**Affiliations:** Department of Endocrinology, Odense University Hospital, 5000 Odense, Denmark; Department of Clinical Research, University of Southern Denmark, 5000 Odense, Denmark; Department of Internal Medicine, John P and Kathrine G McGovern Medical School, University of Texas Health Science Center at Houston, Houston, 77030 TX, United States; Molecular and Human Genetics and Medicine, Baylor College of Medicine and Texas Children's Hospital, Houston TX, TX 77030, United States; Department of Cardiology, Aalborg University Hospital, 9000 Aalborg, Denmark; Kathryn O. & Alan C. Greenberg Center for Skeletal Dysplasias, Hospital for Special Surgery, New York, 10021 NY, United States; Department of Endocrinology and Internal Medicine, Aarhus University Hospital, 8200 Aarhus, Denmark; Centre for Rare Diseases, Pediatric and Adolescent Medicine, Aarhus University Hospital, 8200 Aarhus, Denmark; Department of Medicine, Oregon Health and Science University, Portland, 97239 OR, United States; Department of Endocrinology and Internal Medicine, Aarhus University Hospital, 8200 Aarhus, Denmark; Department of Clinical Medicine, Aarhus University, 8200 Aarhus, Denmark; Practice, Science, and Health Outcomes Research, University of Maryland School of Pharmacy, Baltimore, 21201 MD, United States; Kathryn O. & Alan C. Greenberg Center for Skeletal Dysplasias, Hospital for Special Surgery, New York, 10021 NY, United States; Centre for Genomic and Experimental Medicine, Institute of Genetics and Cancer, Western General Hospital, University of Edinburgh, Edinburgh EH 2XU, United Kingdom; National Jewish Health, Denver, 80206 CO, United States; Alpha-1 Foundation and AlphaNet, Coral Gables, 33134 FL, United States; Department of Pediatrics, Faculty of Medicine at the University of Cologne, University Hospital Cologne, 50937 Cologne, Germany; Division of Orthopedics and Sports Medicine, Children's National Hospital and George Washington University School of Medicine and Health Sciences, Washington, 20010 DC, United States; Department of Medicine, Oregon Health and Science University, Portland, 97239 OR, United States

**Keywords:** osteogenesis imperfecta, collagen type 1, cardiovascular disease, systematic review, delphi process

## Abstract

Osteogenesis imperfecta (OI) is a multisystem disorder most often caused by pathogenic variants in genes that encode type I collagen. Type I collagen is abundant not only in bone but also in multiple tissues including skin, tendons, cornea, blood vessels, and heart. Thus, OI can be expected to affect cardiovascular system, and there are numerous reports of cardiovascular disease (CVD) in people with OI. However, there is no consensus on how CVD in OI should be assessed or managed. To fill this gap, a multidisciplinary group was convened to develop clinical guidance. The work included a systematic review of the available literature and, using a modified Delphi approach, the development of a series of statements summarizing current knowledge. Fourteen clinical recommendations were developed to guide clinicians, patients, and stakeholders about an approach for CVD in adults with OI. This paper describes how the work was conducted and provides the background and rationale for each recommendation. Furthermore, we highlight knowledge gaps and suggest research priorities for the future study of CVD in OI.

## Introduction

Osteogenesis imperfecta (OI) comprises a group of Mendelian disorders that are primarily caused by pathogenic variants in genes encoding α1 or α2 chains of type I collagen, or genes encoding proteins required for post-translational modification, processing, or secretion of type I collagen.^1^^,^^2^ OI can also be caused by pathogenic variants in genes involved in skeletal mineralization or osteoblast function.^1^^,^^2^ The primary phenotypes in OI are fractures and skeletal deformities. However, type I collagen is also an important component of the extracellular matrix of many tissues and thus individuals with OI can present with extraskeletal manifestations.^3^

Type I collagen is abundant in the cardiovascular system,^4–8^ raising the possibility that OI may involve dysfunction of the heart and blood vessels. Studies in some animal models of OI have demonstrated abnormalities in cardiac structure and function. Additionally, there are numerous case reports, cross-sectional studies, and small case series of individuals with OI with cardiovascular diseases (CVD). Population-based analyses have suggested that CVD is more common and/or unique in people with OI. Two systematic literature reviews have summarized the published data on cardiovascular manifestations of OI.^9^^,^^10^ However, there is still an absence of guidelines for appropriate surveillance and management of CVD in OI. The development of guidance is challenging due to the heterogeneity of published literature, predominance of case reports, limitation of sample sizes, low strength of associations, and the nuances involved in interpretation of diagnostic cardiovascular imaging.

Due to the complexity of the topic and the scarcity of available information, we have used a structured consensus development method and re-examined the literature to develop practical clinical recommendations related to cardiovascular issues in adults with OI. The overarching goal was to generate information and provide guidance for patients and families affected by OI, stakeholders including patient advocacy groups, and health care professionals. In addition, we identify existing knowledge gaps and suggest research priorities to promote impactful, patient-centered research on CVD in OI.

## Materials and methods

The project was conceptualized by the Adult OI Working Group, a team of clinician investigators from the U.S. and Europe. By consensus, CVD in OI was considered to be an important issue, with the recognition that there were no existing guidelines for its evaluation or care.

**Figure 1:**
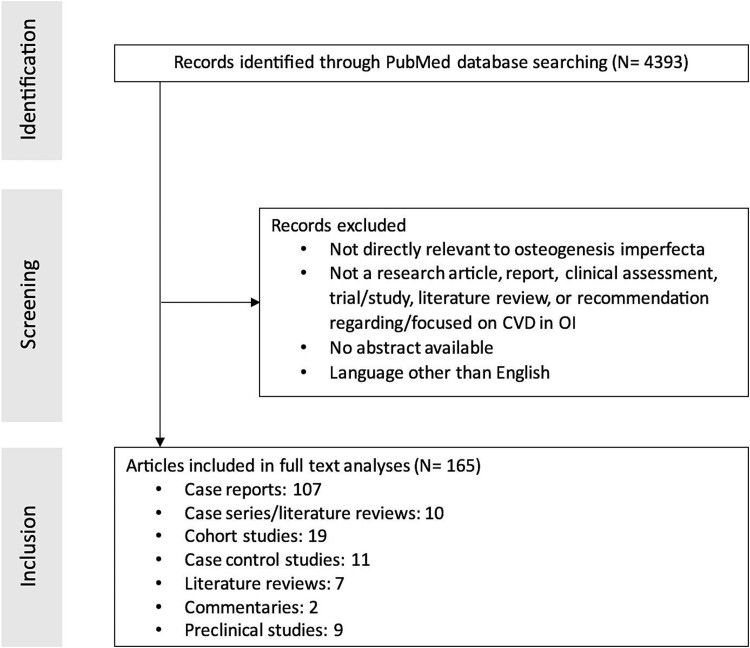
The process of literature and screening.

To generate a comprehensive list of published articles about CVD in OI, a search of PubMed was performed to retrieve studies published between 1960 and 2023. The search process is described in the Supplemental Methods and summarized in Figure 1. The search terms used are listed in Table S2. Overall, 4393 citations were screened by research staff using a prespecified protocol and 165 were deemed to be relevant and appropriate. The convened panel of experts included 9 members of the Adult OI Working Group, and 4 additional clinician investigators (Table S1). An experienced facilitator was recruited to support the process.

A 3-day meeting with rounds of discussion and initial consensus development was held in January 2024. Prior to the meeting, panel members reviewed all the articles and draft statements. During the meeting, those statements were discussed and modified. The final result of 4 rounds of discussions and 3 rounds of voting are presented in this manuscript. The overall approach used had elements of both the RAND/UCLA Delphi and nominal group techniques^11^ and has been more fully described in Supplemental Materials. The results of each voting round can be seen in Tables S3-S5.

## Results

### Statements and clinical guidance

Table 1 includes the 14 statements that summarize background information about CVD in adults with OI as well as its clinical evaluation, management and follow-up. In addition, there are 5 statements identifying gaps in current knowledge and priority directions for future research were also developed.

**Table 1:** Result statements: clinical implications and recommendations.

Section	Statement	Clinical implications and recommendations
**Background of CVD in OI**		
	As in the general population, cardiovascular disease is a major cause of morbidity and mortality in people with OI.	Individuals with OI, providers, and families should be aware that OI is a multisystem disorder, that CVD does occur in OI, and that it may have unique characteristics. Any unexplained symptoms that may have a cardiovascular origin should be promptly evaluated. Evaluation and treatment of CVD in adults with OI should follow clinical guidelines for the general adult population. Current evidence does not support additional screening for CVD in adults with OI unless other indications for CV evaluation, such as chest pain, dyspnea, or clinical symptoms suggestive of heart failure, are not present.
	The biological plausibility of CVD in OI is supported by data from animal models which demonstrate histological, structural, and functional abnormalities in cardiovascular tissues.	Preclinical data suggest that cardiovascular abnormalities could be observed in OI. It is unclear whether, and to what extent, the cardiovascular abnormalities found in preclinical studies are recapitulated in people with OI.
	There is insufficient evidence to confirm that OI type and/or genotype are correlated with risk for and severity of cardiovascular disease.	Stratification or risk assessment regarding CVD in OI cannot be made based on the genotype in an individual with OI. Possibility of cardiovascular complications should be considered in the care of all patients with OI, regardless of genotype.
	Myocardial structural and biomechanical abnormalities that affect cardiac function have been reported in people with OI; however, the clinical implications of those abnormalities are not well understood.	Currently available evidence does not support routine screening for structural heart disease in adults with OI Currently available evidence does not support the initiation of medical therapy in individuals who are found to have changes in cardiac structure without impairment of cardiac function. Evaluation and treatment of cardiac structure or cardiac function in adults with OI should be consistent with current clinical guidelines for all adults with suspected CVD
**Clinical evaluation of CVD in OI**		
	Shortness of breath as a symptom is more common in people with OI than in the rest of the population. It is not clear whether this is due to cardiovascular, pulmonary, or other causes.	As dyspnea can stem from multiple causes, clinicians must be vigilant in identifying the origin of dyspnea, including an evaluation of cardiovascular and pulmonary systems.
	Cardiac valvular abnormalities, particularly mitral and/or aortic valve regurgitation, appears to be common in people with OI, however, the clinical implications are not well understood.	Clinicians should be aware of the association between OI and both aortic and mitral regurgitation and recognize that underlying defects in type I collage might contribute to valvular abnormalities. Adults with OI should be examined clinically for evidence of valvular disease. Current evidence does not support the use of routine echocardiographic screening for valvular disease in adults with OI who do not have symptoms suggestive of valvular disease. If clinical evaluations suggest the possibility of valvular disease, echocardiography should be performed, supplemented, where appropriate, by other imaging. If clinically significant valvular abnormalities are found, referral to a cardiologist is recommended. When possible, CVD in OI should be managed in specialized high-volume centers, where they can receive ongoing monitoring from experts in valve disorders.
	Aortic root dilation appears to be more common in people with OI, however the clinical implications of this dilation are not well understood.	Evaluation of aortic disease in adults with OI should be consistent with clinical guidelines for the general adult population. Current evidence does not support routine screening for aortic dilation in adults with OI if other indications for cardiovascular imaging, such as valvular heart disease, stroke, surveillance after aortic operations, or suspected arterial dissection, are not present. Currently evidence does not support that initiation of medical therapies can slow or prevent aortic dilation in adults with OI. Clinicians should be aware that indexed aortic root sizes in people with OI may be affected by short stature.
	Vascular aneurysms and dissections have been reported in people with OI, however, the overall prevalence of aneurysms is unknown and the risk for dissections is not well understood.	Routine evaluation of arterial disease in adults with OI should be consistent with clinical guidelines for the general adult population. Vascular dissections can occur in adults with OI, but it is not known if the frequency is increased in comparison with the general population. Current evidence does not routine support screening for cervical or cerebral aneurysms in adults with OI Unexplained chest, back, or leg pain, or other symptoms such as focal neurologic deficits that do not resolve spontaneously should be investigated and a vascular cause should be sought.
	Baseline and periodic clinical cardiovascular evaluations are appropriate in all adults with OI. If abnormalities are identified, referral to a cardiovascular specialist should be considered.	There appears to be an increase in cardiac failure and valve disease in OI after the age of approximately 50 yr, and clinicians should be especially alert for clinical signs of CVD in older people. Evaluations of adults with OI should include a routine clinical evaluation of the cardiovascular system. Inquiry about specific symptoms, such as shortness of breath or palpitations, and important elements of a cardiac examination, such as auscultation for murmurs, pulses, edema, and peripheral signs of aneurysms or dissections, should be emphasized.
	Automated blood pressure cuffs can be used in people with OI. However, caution should be exercised in examining people with severe OI and/or skeletal deformity.	Automated blood pressure cuffs can generally be safely utilized in adults with OI. In individuals with severe deformities of arms, careful consideration and potentially alternative methods may be necessary to ensure accurate and safe blood pressure measurements.
	Techniques for echocardiographic evaluation of adults with OI should be adapted to the individual’s body shape and size.	Currently, there are no specific echocardiographic protocols for adults with OI and thus standard procedures should be followed when reporting cardiac structure and function. Due to the suspected increased frequency of aortic dilation in OI, the thoracic aorta should also be measured at four locations (annulus, sinuses of Valsalva, mid-ascending, and arch) using the leading edge to leading edge technique. When requesting an echocardiogram, clinicians and patients should specify that these four aortic diameters are to be reported. Cardiac and aortic measurements should be adjusted to body size by indexing or *Z*-scores. Whenever possible, evaluations should occur at centers with particular expertise and experience in the ultrasound evaluation of congenital heart disease or aortic disease.
**Surgical Management of CVD in OI**		
	Based on limited evidence from case reports, aortic valve and mitral valve surgeries can be successful in people with OI.	To optimize surgical outcomes, clinical management should involve a multidisciplinary approach and careful perioperative preparation Whenever possible, surgery should be performed in high-volume centers that have expertise in complex cardiac surgeries.
	Although the literature is limited to case reports and small case series, cardiac surgery has been reported to have a greater risk of complications in people with OI.	Tissue fragility, bleeding, and poor wound healing may be more likely in adults with OI. If surgery for cardiovascular disease is indicated, special attention to these issues is appropriate in the peri-operative period. Due to the risk of surgical bleeding complications, drugs and equipment to ensure hemostasis should be available in the OR.
	When people with OI undergo cardiovascular surgery particular care should be taken to optimize surgical outcomes and minimize complications.	When clinically indicated, cervical spine imaging should be performed Special attention should be paid to pulmonary and anesthesia-related concerns Careful positioning on the operating table with padding and support of the neck and extremities are recommended Whenever possible, surgical procedures should be pursued at facilities with awareness of the overall clinical features of OI and which can evaluate and manage potential risks and complications. For both elective and urgent surgeries, it is essential to plan and share knowledge, experience, and expectations among the multidisciplinary team and with the patient. While emergency circumstances may preclude ideal pre-operative planning and shared decision-making, these clinical guidelines should inform the management of both elective and emergency surgeries.
**Gaps in current knowledge and research priorities**		
	The current evidence related to CVD in OI is primarily limited to case reports, small case series and cross-sectional studies, which introduces publication and selection bias. To better understand the nature of cardiovascular abnormalities in people with OI, detailed cellular and molecular studies in preclinical models and human tissues are needed. To determine the prevalence, types and outcomes of CVD in people with OI, cardiovascular and genetic evaluations of large cohorts of the OI population worldwide (recruited in an unbiased manner) are needed, with selection of appropriate controls. To better understand the progression of cardiovascular abnormalities in people with OI, longitudinal studies are needed to evaluate cardiovascular function and outcomes across the lifespan. Potential differences due to sex and race/ethnicity should be studied. Future research will be strengthened if conducted in a patient-centered way, with active participation and input from people with OI and other stakeholders Future endeavors should develop resources for healthcare providers and people with OI that would facilitate appropriate evaluation and treatment of cardiovascular disease.	

Abbreviations: CVD, cardiovascular diseases; OI, osteogenesis imperfecta.

### Literature summaries (references for this section can be found in Supplemental Material)

#### Background of CVD in adult OI

1. As in the general population, CVD is a major cause of morbidity and mortality in people with OI.

In Danish OI cohort studies,^12^^,^^13^ CVD represented the most common cause of death in OI, occurring in 2.9% of the OI cohort (accounting for 17.9% of all deaths in OI) and in 1.9% of the control group (accounting for 25.6% of all deaths in controls). The difference between OI and control groups was not statistically significant (HR 1.5 [CI, 0.9-2.5]). In 79 people with OI from the UK, McAllion and Paterson^14^ evaluated the causes of death based on data from death certificates, postmortem reports, medical records, hospital consultants, relatives, and data from the Brittle Bone Society and found that cardiovascular causes of death were observed in 36.6% of people with OI types I and IV, and 2.6% of people with OI type III as compared with 44.6% of people in the general population. Hence, similar to the overall population, deaths due to CVD were common in OI. In the Danish cohort studies,^12^^,^^13^ a diagnosis of heart failure was significantly more common in OI cohort compared to reference population (HR 2.3 [95% CI, 1.4-3.7], *p* < .001). The increased risk persisted even after adjusting for confounders like the presence of ischemic heart disease (HR 2.4 [95% CI, 1.5-3.8], *p* < .001). Moreover, the age at onset of heart failure was significantly lower in the OI population compared to the reference population (58 yr [95% CI, 44-69] vs 76 yr [62-83], *p* < .001). In those studies,^12^ the prevalence of ischemic heart disease was similar in the OI cohort and the reference population. Furthermore, the prevalence of risk factors for CVD such as diabetes and hyperlipidemia were similar, but treatment with antihypertension medications was more common in the OI group (28.1% vs 21.6%, *p* < .0001) as was the use of non steroidal anti-inflammatory drugs (NSAIDs) (56.6% vs 47.2%, *p* < .001).

1. The biological plausibility of CVD in OI is supported by data from animal models which demonstrate histological, structural, and functional abnormalities in cardiovascular tissues.

Type I collagen is a major extracellular matrix constituent of the cardiac myocardium, chordae tendinae, and cardiac valves.^5–8^^,^^15–18^ Furthermore, fibrillar collagens, that is, types I and III, are important components of the walls of arteries including the aorta.^19^^,^^20^ Cardiovascular abnormalities have been demonstrated in two murine models with *Col1a1* pathogenic variants (*Col1a1^Aga2/+^* and *Col1a1^Jrt/+^*) and one model with a *Col1a2* variant (oim). The *Col1a1^Aga2/+^* model has varying severity of phenotypes^21^ and mice with a more severe phenotype have disorganized matrix collagen with fewer and thinner collagen fibrils in cardiac tissues, enlarged interventricular septa, right ventricular hypertrophy, decreased fractional shortening of the left ventricle, and lower ejection fraction compared to wild type littermate control mice.^22^ The *Col1a1^Jrt/+^* mouse model, a combined model for OI and Ehlers-Danlos Syndrome, has myocardial hypertrophy.^23^ Homozygous *oim* mice have a severe nonlethal form of OI and demonstrate smaller myocardial collagen fiber area fraction, lower collagen fiber number and diameter, and abnormalities in left ventricular volume.^24^ Furthermore, homozygous *oim* mice have reduced collagen content, altered collagen crosslinking and biomechanical integrity of aorta,^25^ proteomic changes and disturbances on extracellular homeostasis in aortic valves, and thickening of aortic valves with age.^26^ To our knowledge, cardiovascular phenotypes have not been published in other commonly used mouse models of OI (ie, *Col1a1^m1Btlr^* and *Col1a2^G160C/+^*).

1. There is insufficient evidence to confirm that OI type and/or genotype are correlated with risk for and severity of CVD.

While there is evidence to show an association between genotype and severity of skeletal manifestations in OI,^27^ similar associations between genotype and CVD in OI are lacking. Some publications have posited that cardiovascular symptoms may be more common in individuals with OI caused by quantitative defects in type I collagen.^28^^,^^29^ Whereas some case series report no evidence for a correlation between the disease-causing genes *COL1A1* and *COL1A2* and CVD, others report a higher frequency of cardiovascular abnormalities in individuals with OI caused by variants in *COL1A1* compared to *COL1A2.*^30^^,^^31^ However, this literature is very limited and based on small case series without statistically significant genotype – phenotype correlations.

1. Myocardial structural and biomechanical abnormalities that affect cardiac function have been reported in people with OI; however, the clinical implications of those abnormalities are not well understood.

Two case series and three echocardiographic studies have documented structural cardiovascular changes in humans with OI.^32^^–^^37^ However, there were minimal changes in left ventricular ejection fraction or other measures of left ventricular systolic function. Weis et al. hypothesized that increases in left ventricular internal diameter and wall thickness may be a compensatory mechanism for reduced myocardial type I collagen.^24^ Two studies have identified right ventricular dilation and reduced right ventricular systolic function in an adult Norwegian cohort comprising 47 individuals with OI.^35^^,^^38^ These studies and one other case–control study also documented mildly decreased left ventricular diastolic functional parameters in adults with OI, corresponding to stage 1 diastolic dysfunction.^33^ There is currently insufficient evidence to conclude that cardiac changes in most adults with OI are clinically significant or that such changes progress meaningfully over time.

#### Clinical evaluation of CVD in OI

1. Shortness of breath as a symptom is more common in people with OI than in the rest of the population. It is not clear whether this is due to cardiovascular, pulmonary, or other causes.

Pulmonary issues and respiratory failure represent a major cause of death in individuals with OI,^12^^,^^14^^,^^39^^–^^41^ and people with OI have a high prevalence of shortness of breath. In a Norwegian study on the prevalence of symptoms of CVD,^38^ 19% of 47 individuals with OI reported dyspnea. Shortness of breath was highlighted as a major concern in a focus group study as part of the KEY4OI Plus Pulmonary.^42^ The IMPACT survey, which included including 2988 individuals with OI and their caregivers, revealed that “lung and breathing problems” were frequent in the adult OI population.^43^ While a cardinal manifestation of heart failure is dyspnea,^44^ the extent to which cardiac dysfunction contributes to pulmonary symptoms in OI is unclear.

1. Cardiac valvular abnormalities, particularly mitral and/or aortic valve regurgitation, appear to be common in people with OI; however, the clinical implications are not well understood.

There are numerous case reports of cardiac valve disorders in adults with OI. Ashournia et al.^9^ reviewed the existing body of case reports and small series and found that 59 individuals with OI were reported with valve abnormalities. They concluded that echocardiographic valve abnormalities are likely to be more common in people with OI than in the general population. Aortic valve insufficiency appears to be most frequently reported valvular abnormality; however, several individuals with mitral regurgitation and individuals with both aortic and mitral regurgitation have also been reported. Clinically important right-sided valve disease has rarely been described. Some case reports include histological studies of valve tissue obtained at the time of surgical intervention,^34^ and a variety of left-sided histological valvular abnormalities including myxomatous degeneration, cystic medial necrosis, and/or disorganized collagen architecture have been described.

The Danish OI cohort studies have shown that mitral regurgitation and aortic regurgitation are significantly more common in the OI population than in population-based controls (HR = 6.3 [2.5-15.5] and HR = 4.5 [1.4-13.9, respectively).^13^ There appears to be an overemphasis on the prevalence of congenital cardiac anomalies in case reports of individuals with OI, without an apparent increase compared to the general populace.^45^ Notably, valve abnormalities that apparently have no clinical implications are commonly observed in studies of the general population,^46^ and thus the significance of these findings in OI may be exaggerated.

Although longitudinal evaluations of cardiac health within OI cohorts are lacking, the clinical diagnosis of valve disease in people with OI typically occurs in those over 40 yr of age for aortic valve regurgitation and over 60 yr for mitral valve disease, with a relatively small proportion of individuals needing intervention for such valvular abnormalities.^13^

1. Aortic root dilation appears to be more common in people with OI; however, the clinical implications of this dilation are not well understood.

The frequency of proximal thoracic aortic dilation in adults with OI is difficult to estimate precisely because the methods used to assess aortic dilation and the composition of study cohorts have been variable. Two case series from the 1980s that included people with several types of OI^32^^,^^47^ reported aortic root dilation (involving the annulus, sinuses of Valsalva, sinotubular junction, and/or proximal ascending aorta) in 12%-30% of individuals. More recent studies, primarily comprising people with OI type I, reported small (1-2 mm) but statistically significant differences in aortic diameters compared to matched controls, without identifying a threshold value for aortic dilation.^9^^,^^36^^,^^37^ In the absence of severe aortic regurgitation or hypertension, clinically significant aortic dilation (defined by an indexed aortic diameter >2.0 cm/m^2^ of body surface area or a *Z*-score > 3) appears to be rare in adults with OI. There are no longitudinal data about how aortic diameters change over time.

1. Vascular aneurysms and dissections have been reported in people with OI; however, the overall prevalence of aneurysms is unknown and the risk for dissections is not well understood.

In OI, vascular collagen fiber content may be reduced, and collagen crosslinking may be abnormal.^4^ Cerebral arteries may be vulnerable to rupture in OI because their tensile strength is primarily derived from adventitial collagen.^48^ Almost all case reports about spontaneous dissections in adults with OI describe cerebral arterial dissections or cervical arterial dissections involving intracranial segments of carotid or vertebral arteries causing stroke or death due to embolism or subarachnoid hemorrhage.^48^^–^^58^ However, cerebral aneurysms predisposing to subarachnoid hemorrhage are relatively common in the general population (0.5%-3%).^54^^,^^59^ From the current literature, it is not clear whether OI predisposes to the formation of intracranial aneurysms or increases the likelihood that an aneurysm will rupture or dissect. Thoracic or abdominal arterial aneurysms or dissections have rarely been reported in adults with OI.^58^^,^^60^^,^^61^ While preclinical data from homozygous *oim* mice have shown reduced levels and crosslinking of collagen in aortas which were prone to rupture when pressurized to supraphysiologic levels, spontaneous aortic dissection or rupture appears to be rare in humans with OI.^25^ Dissections appear to be more likely to occur after trauma or previous aortic interventions such as aortic valve replacement.^14^^,^^59^^,^^62^^–^^64^

The overall frequency of arterial aneurysms and dissections in adults with OI is difficult to estimate precisely because arterial imaging and clinical symptoms related to arterial events have not been systematically documented in large cohorts. In the Danish OI cohort studies, the reported frequency of vascular dissections or aneurysms in adults with OI was not increased compared to the general population.^13^

1. Baseline and periodic clinical cardiovascular evaluations are appropriate in all adults with OI. If abnormalities are identified, referral to a cardiovascular specialist should be considered.

As previously discussed, type 1 collagen fibers are present in several components of the heart, contributing to maintenance of cellular architecture and passive as well as active cardiac properties.^33^^,^^67^^,^^68^ A Norwegian observational study presented findings from a comprehensive cardiac assessment of 99 adults with OI who had no known CVD.^38^ Despite observing only minor and clinically insignificant changes in blood pressures, electrocardiograms, and echocardiograms, including tissue velocity imaging, more than one-third with OI reported experiencing chest pain and one-fifth reported shortness of breath.^38^ Symptoms that may suggest impaired cardiac function or vascular abnormalities should prompt a thorough evaluation to determine their etiologies.

1. Automated blood pressure cuffs can be used in people with OI. However, caution should be exercised in examining people with severe OI and/or skeletal deformity.

People with OI may have increased health care needs, and hence blood pressure monitoring may be more frequent. While there is concern about increased susceptibility to bruising and fractures from using blood pressure cuffs, the existing literature is sparse, with only a few reports addressing this issue. A small study of 37 children found no risk of upper arm fractures associated with automated blood pressure cuffs.^69^ Similarly, a retrospective study reporting 83 patients and 205 anesthetic events over 7 yr reported no complications from blood pressure assessment.^70^ In adults, a cohort study involving 273 surgical procedures, including 229 orthopedic interventions, did not identify major issues with automated blood pressure cuffs. In summary, the available evidence suggests that there should be no substantial concern for upper arm fractures related to the use of automated blood pressure determinations when appropriately performed.

1. Techniques for echocardiographic evaluation of adults with OI should be adapted to the individual’s body shape and size.

In adults with OI who have chest wall deformities, complete imaging of the heart and aorta using echocardiography may be challenging and frequently requires alternative thoracic imaging planes or additional subcostal views.^71^ As many adults with OI are significantly smaller than age- and sex-matched controls, using absolute size thresholds based on general population norms to classify ventricular or aortic dilation in OI may decrease or delay recognition of abnormalities. Indexing aortic or ventricular dimensions to body size can correct for this size difference. The most frequently used indexing methods for the aorta are the *Z*-score (dimensionless unit indicating the number of standard deviations from the population mean), the height index (absolute diameter in millimeters divided by body length in meters), and the size index (absolute length in centimeters divided by body surface area (BSA) in square meters).^72^  *Z*-scores are typically adjusted for age, sex and BSA using multivariable linear regression.^73^ The size index method is also frequently used to adjust ventricular dimensions and the calculated left ventricular mass.^74^ Size indices and *Z*-scores may not be accurate for adults with OI who are more than two standard deviations from the population mean BSA (for women: 1.5-2.2 m^2^; for men: 1.7-2.5 m^2^).^75^ There is currently insufficient evidence to recommend one indexing method over another. It may be useful to compare more than one method. Confirmatory studies are needed to clarify which indexing methods may be optimal for adults with OI.

#### Surgical management of CVD in OI

1. Based on limited evidence from case reports, aortic valve and mitral valve surgeries can be successful in people with OI.

Evidence about surgical outcomes in OI is limited and is largely based on case reports or small case series.^34^ Cardiac operations are primarily reported in older adults with OI (ie, age greater than 60 yr), but younger adults with bicuspid aortic valve and other congenital valve abnormalities have also been included in some case series.^66^

The risk of complications associated with cardiovascular surgery in people with OI has been reported to be high. Aoki et al.^76^ found a mortality rate of 18% (6/33). The authors stipulate that capillary fragility and platelet dysfunction could play an important role in this increased risk. On the other hand, a number of case reports and small case series have reported positive outcomes after cardiovascular surgery in OI.^76^^–^^119^ Similarly, although morbidity and mortality risks associated with valve surgery in OI are reported to be increased,^120^ the overall rate of cardiovascular mortality after valve surgery does not appear to be higher compared to the general population, implying generally favorable outcomes.^13^ The rate of reoperation for recurrent valve regurgitation after valve repair in adults with OI is not currently understood.^83^

For valve replacements, bioprosthetic valves were proposed to be a safer option than mechanical valves for adults with OI due to surgical challenges associated with tissue fragility and increased bleeding risks.^34^^,^^65^ Moreover, in individuals who may require orthopedic procedures where valve thrombosis or bleeding could be a problem, bioprosthetic valves and the resulting avoidance of lifelong anticoagulation could be especially beneficial.^65^

1. Although the literature is limited to case reports and small case series, cardiac surgery has been reported to have a greater risk of complications in people with OI. Tissue fragility, bleeding, and poor wound healing may be more likely.

In one study from the Netherlands,^121^ 18% of the 23 participants had increased bleeding score using the bleeding assessment tool created by the International Society on Thrombosis and Haemostasis. Moreover, while there are currently no structured observational studies of wound healing in OI, clinical experience and patient-reported data indicate that wound healing may be abnormal in people with OI. In a study by Hansen et al.,^122^ evaluating the mechanical properties of skin in 10 patients with OI, all had decreased elasticity, distensibility, and hysteresis compared with controls. These changes differ from age-related changes, which have been described as increased distensibility and viscosity (like hysteresis).

1. When people with OI undergo cardiovascular surgery, particular care should be taken to optimize surgical outcomes and minimize complications.

OI is a pleiotropic disorder, and people with OI may have surgical risks unrelated to the cardiac problem at hand that require systematic pre-surgical evaluation to facilitate perioperative planning. Cervical spine and craniocervical abnormalities are known features of OI,^123^^–^^127^ and pre-operative imaging, such as spine X-rays with dedicated views of the cervical spine, may help direct management. Patients with any neurologic signs and symptoms may require treatment prior to undergoing any type of surgery, and at a minimum should be managed with cervical spine precautions in the operating room. Careful pre-surgical anesthesia airway assessment and planning of the anesthetic approach are recommended.^128^^–^^131^ Sleep apnea is reported in up to 50% of people with OI, and this may have implications for anesthesia and airway management.^132^^–^^136^ Particular attention to bone fragility and the risk of fracture is essential. Care should be exercised during patient transfers and positioning in the OR. Recommendations include padding of spine and extremities, especially in the setting of deformity and contractures.^137^

#### Gaps in current knowledge and research priorities

1. The current evidence related to CVD in OI is primarily limited to case reports, small case series and cross-sectional studies, which introduces publication and selection bias.

The evidence related to CVD in OI is at high risk of bias. In rare disorders, case reports may serve as the sole means to highlight complications. As rare and unusual presentations are more likely to be published than common presentations, generalizing the information from case reports is difficult, and such reports don’t help in establishing cause-effect relationships. Patient ascertainment bias and selection bias may limit the generalizability of the findings from small series.^34^ Since the clinical presentation of people with OI is so variable, the difficulty in assembling adequately representative study groups is a particular problem.

1. To better understand the nature of cardiovascular abnormalities in people with OI, detailed cellular and molecular studies in preclinical models and human tissues are needed.

Whereas some studies in preclinical models of OI have shown evidence for abnormal structure and or function of the heart and blood vessels, the body of work exploring the mechanistic basis for such abnormalities, and therapeutic approaches that can be used to alleviate cardiovascular manifestations, remain limited. The models in which CVD has been demonstrated, that is, *Col1a1^Jrt/+^*, *Col1a1^Aga2/+^*, and *oim* mice, are not representative of the manifestations of OI in most humans. The majority of individuals with OI have OI types I and IV and the cardiovascular manifestations in preclinical models of these OI types have not been studied. Moreover, the consequences of cell- and tissue-specific abnormalities of type I collagen in the heart and vasculature has not been examined. Thus, additional animal models that more closely mirror human OI phenotypes would be pivotal for evaluating the pathophysiology of CVD in OI and potential diagnostic and therapeutic approaches.

1. To determine the prevalence, types, and outcomes of CVD in people with OI, cardiovascular and genetic evaluations of large cohorts of the OI population worldwide (recruited in an unbiased manner) are needed, with selection of appropriate controls. In addition, to better understand the progression of cardiovascular abnormalities in people with OI, longitudinal studies are needed to evaluate cardiovascular function and outcomes across the lifespan. Potential differences due to sex and race/ethnicity should be studied.

Most current knowledge about the cardiovascular manifestations of OI is derived from case reports, case series, and single-center cross-sectional studies. More effective study designs are needed. Some of the key questions that should be addressed include: (1) prevalence and natural history of CVD in individuals with OI, (2) risk factors for development of CVD in OI, (3) appropriate surveillance measures to monitor for CVD in OI including the frequency of such monitoring, and its efficacy in improving long-term outcomes, and (4) outcomes of medical and surgical interventions to treat CVD in OI. Adequately powered longitudinal epidemiological and clinical studies are essential to define the nature, prevalence, and genetics of CVD in OI.

Those studies should adequately represent the spectrum of clinical and genetic heterogeneity in OI, should include children and adults of both sexes, and have individuals with different races and ethnicities. Since aging has significant effects on CVD, studies must also include older individuals.

1. Future research will be strengthened if conducted in a patient-centered way, with active participation and input from people with OI and other stakeholders.

Future studies will be strengthened by centering patients in the research process through engagement of people with OI, caregivers, and other stakeholders. They can inform research questions based on their lived experiences, and in collaboration with OI researchers can co-develop research strategies, protocols, patient facing materials, and methods for communicating results. People with OI may be more likely to participate in longitudinal observational studies and clinical trials if the study objectives are aligned with their priorities. The overall goal of these studies will be to help patients, families, and clinicians make informed treatment decisions to improve the quality of life for people with OI.

1. Future endeavors should develop resources for healthcare providers and people with OI that would facilitate appropriate evaluation and treatment of CVD.

A frequent challenge for patients with rare disorders and their health care providers is having access to information that is necessary to optimize care. This has been the situation with OI, especially since most patients and providers are not linked to tertiary care medical centers that have specialized expertise. Thus, it would be beneficial to develop methods for the effective dissemination of clinically useful information about cardiovascular involvement in OI, in forms that are targeted to each audience. Patient advocacy organizations continue to play an important role in connecting both patients and providers to credible resources, acting as trusted sources of information, and as clearinghouses of medically verified, evidence-based information that is accessible. Institutions with centers of excellence in OI can similarly develop and disseminate useful information, including recent research findings that have clinical application, and can provide readily accessible portals for inquiries about cardiovascular care.

## Discussion

The available data about CVD in OI are complex, limited, and inconsistent. There are no practical guidelines to assist people with OI and their clinicians in the assessment and care of cardiovascular problems. Our summaries should provide context for considering the type of cardiovascular problems that may occur, how they may present in clinical settings, and what interventions have been described. This information should be helpful in considering the impact of cardiovascular disorders in the OI population, as well as at an individual, patient-by-patient level.

We have attempted to develop practical guidance for the care of cardiovascular issues in people with OI. While these recommendations are unavoidably limited by the scarcity of the existing information, they allow for more informed care of individuals with OI. This work also provides a framework for developing institutional or community strategies for the care of people with OI. Finally, this work offers information and perspective for individuals with OI who are concerned about their personal risk of cardiovascular problems.

A major objective was to develop recommendations for critical research goals. The literature about cardiovascular manifestations in OI is dominated by case reports and relatively small observational series. Whereas such information is useful, it is subject to significant bias. Additional research is necessary to provide confidence about the nature of cardiovascular involvement in OI. Studies of large cohorts of people with OI are needed to better determine the prevalence, incidence, and characteristics of cardiovascular involvement. In addition to clinical research, basic and translational studies are needed to better understand the pathophysiology of the cardiovascular manifestations for OI. Finally, patient engagement in the planning and conduct of research is necessary to ensure completeness and relevance.

This work offers a unique addition to the existing literature about OI. We used a carefully planned and organized method to develop consensus among a group of experts with diverse backgrounds to synthesize the available information to yield practical guidance. Despite these strengths, our approach had some limitations. The group of experts was relatively small, and the effort may have benefited from a larger, more diverse panel of experts. We did not formally include patient involvement. The process was inherently restricted by the scarcity and nature of the available information about cardiovascular disturbance in OI. While we used a well-designed Delphi approach involving several iterative rounds of structured discussion, our interactions during consensus development were not anonymized, potentially introducing some unintended influences.

In summary, we comprehensively reviewed the available information about CVD in adults with OI and developed a set of observations and clinical recommendations that can be helpful in understanding and managing cardiovascular issues, identified critical knowledge gaps, and have suggested research directions that are necessary to address them.

## Supplementary Material

Supplement_Table_1_Panel_Members_zjae197

Supplement_Table_2_pubmed_search_terms_zjae197

Supplement_Table_3_First_round_of_votes_zjae197

Supplement_table_4_Second_round_of_votes_zjae197

Supplement_table_5_Final_round_of_votes_zjae197

Supplement_1_Methods_zjae197

Supplement_2_Full_list_of_references_zjae197

## Data Availability

The data that support this study’s findings are openly available via the reference list and Supplement Material available for this paper. The statements agreed upon as described in this paper were reached via discussion. There are no recordings available from this meeting, but the conclusions are based on the current literature as referenced here. We are always open to discussion, and any interested parties can contact the corresponding author for additional information about how the conclusions were reached.
