# Supplementary material for: Cardiovascular disease in adults with osteogenesis imperfecta: clinical characteristics, care recommendations, and research priorities identified using a modified Delphi technique
Source: J Bone Miner Res. 2024 Dec 12;40(2):211–21. doi: 10.1093/jbmr/zjae197 (PMC11789389; doi:10.1093/jbmr/zjae197)
Supplement: Supplement_Table_1_Panel_Members_zjae197 [file supplement_table_1_panel_members_zjae197.docx]

**Supplement Table 1. Members of the panel.**

| **Panel Member** | **Institution** | **Specialty** |
| --- | --- | --- |
| Erin Carter | Hospital for Special Surgery, New York, NY, USA | Genetic counseling |
| Lars Folkestad | Odense University Hospital, Odense Denmark | Endocrinology |
| Jannie Dahl Hald | Aarhus University Hospital, Aarhus, Denmark | Endocrinology |
| Niels Holmark Andersen | Aalborg University Hospital, Aalborg, Denmark | Cardiology |
| Bente Landahl | Aarhus University Hospital, Aarhus, Denmark | Endocrinology |
| Sandesh C.S. Nagamani | Baylor College of Medicine, Houston, TX, USA | Genetics |
| Eric Orwoll | Oregon Health & Science University, Portland, OR, USA | Endocrinology |
| Siddharth K. Prakash | University of Texas Health Science Center at Houston, TX, USA | Cardiology |
| Cathy Raggio | Hospital for Special Surgery, New York, NY, USA | Orthopedics |
| Stuart H. Ralston | University of Edinburgh, Edinburgh, Scotland | Endocrinology |
| Robert Sandhaus | National Jewish Health, Denver, CO, USA | Pulmonary |
| Oliver Semler | University Cologne, Cologne, Germany | Endocrinology |
| Laura Tosi | Children’s National Hospital, Washington D.C., USA | Orthopedics |
