# Supplementary material for: Cardiovascular disease in adults with osteogenesis imperfecta: clinical characteristics, care recommendations, and research priorities identified using a modified Delphi technique
Source: J Bone Miner Res. 2024 Dec 12;40(2):211–21. doi: 10.1093/jbmr/zjae197 (PMC11789389; doi:10.1093/jbmr/zjae197)
Supplement: Supplement_Table_2_pubmed_search_terms_zjae197 [file supplement_table_2_pubmed_search_terms_zjae197.docx]

**Supplement Table 2. Pub Med search terms used in the initial stage in in the subsequent stage of statement refinement**

| **Search terms - initial stage** | **Search terms - statement refinement** |
| --- | --- |
| osteogenesis imperfecta | surgery osteogenesis imperfecta complications risks |
| cardiovascular disease in osteogenesis imperfecta | surgery osteogenesis imperfecta risks |
| cardiac disease in osteogenesis imperfecta |  |
| vascular disease in osteogenesis imperfecta |  |
| cardiovascular osteogenesis imperfecta adult |  |
| heart osteogenesis imperfecta adult |  |
|  |  |
