# Supplementary material for: Cardiovascular disease in adults with osteogenesis imperfecta: clinical characteristics, care recommendations, and research priorities identified using a modified Delphi technique
Source: J Bone Miner Res. 2024 Dec 12;40(2):211–21. doi: 10.1093/jbmr/zjae197 (PMC11789389; doi:10.1093/jbmr/zjae197)
Supplement: Supplement_Table_3_First_round_of_votes_zjae197 [file supplement_table_3_first_round_of_votes_zjae197.docx]

**Supplement Table 3. Results of the first round of panel voting**

| **Round 1** |  |  |  |  |  |
| --- | --- | --- | --- | --- | --- |
| **Statement** | **Strongly Agree** | **Agree** | **Neutral** | **Disagree** | **Strongly Disagree** |
| Cardiopulmonary disease is the number one cause of death in people with OI and occurs earlier in life than the average population. | 4 | 2 | 3 | 2 | 0 |
| Genetic confirmation of OI type is an important test to inform cardiac risk in the OI population. | 0 | 3 | 4 | 4 | 0 |
| Cardiomyocytes in people with OI have an impaired stress response as an adaptation to low left-ventricle stiffness. | 2 | 4 | 4 | 1 | 0 |
| In people with OI, there are myocardial structural and biomechanical abnormalities that contribute to deficits in cardiac function. | 4 | 6 | 0 | 1 | 0 |
| Cardiac valve disorders, particularly mitral and aortic insufficiency, are more common in people with OI. | 5 | 4 | 1 | 1 | 0 |
| Individuals with more severe OI (type III) are more likely to have cardiac valvopathies than people with less severe types of OI (types I and IV). | 0 | 4 | 5 | 1 | 1 |
| Cardiac valve abnormalities in people with OI appear to be due to abnormal ECM structure, myxomatous changes, and/or cystic medial necrosis. | 1 | 7 | 2 | 1 | 0 |
| Aortic root dilation is commonly found in people with OI. | 1 | 3 | 3 | 4 | 0 |
| Vascular aneurism and dissection may be more likely to happen in people with OI than those without OI. | 2 | 3 | 1 | 5 | 0 |
| Automated blood pressure cuffs should not be used in people with OI. | 2 | 1 | 3 | 5 | 0 |
| All adults over the age of eighteen with OI should have an annual EKG and ECHO. | 1 | 0 | 1 | 7 | 2 |
| Periodic screening with cardiac echocardiography is appropriate in asymptomatic adults with OI. If abnormalities are discovered, repeat echo examinations at 3-5yr intervals. If no abnormalities are discovered, repeat echo evaluation at 10yr intervals. | 1 | 5 | 2 | 2 | 1 |
| Techniques for ECHO evaluation should be adapted to the patient's body shape and must be based on BSA. | 5 | 5 | 1 | 0 | 0 |
| MRI or CT scan with contrast angiography of the heart should be done for complete evaluation of ECHO-positive patients. | 2 | 3 | 4 | 2 | 0 |
| Adults with OI should have cardiac catheterization, which includes right-side heart evaluation. | 0 | 1 | 0 | 7 | 3 |
| Mitral-valve prolapse occurs more frequently in people with OI than in the average population. | 4 | 3 | 3 | 1 | 0 |
| Although the literature is limited to case reports or very small case series, aortic- and mitral-valve replacements can be successful in people with OI. | 1 | 10 | 0 | 0 | 0 |
| Although the literature is limited to case reports or small case series, cardiac surgery has been reported to have a greater risk of complications in people OI than those without OI. Tissue fragility, bleeding and poor wound healing may be more likely. | 2 | 8 | 1 | 0 | 0 |
| All people with OI undergoing anesthesia should have cervical spine evaluation by radiographs and MRI as indicated by x-rays. | 2 | 2 | 5 | 2 | 0 |
| Cardiac surgery in people with OI should be well-planned preoperatively by a multidisciplinary team. | 7 | 4 | 0 | 0 | 0 |
| For people with OI undergoing surgery, there should be careful positioning of the patient in the operating room with padding and support of the extremities. | 8 | 3 | 0 | 0 | 0 |
| TAVR/TMVR may be an alternative to an open sternotomy for people with OI. | 2 | 4 | 4 | 1 | 0 |
| Due to the risk of surgical bleeding complications in people with OI, hemostatic glues, antifibrinolytic agents and blood products including recombinant factor VIIA should be available in the OR. | 2 | 6 | 3 | 0 | 0 |
| To understand the nature of cardiovascular abnormalities in people with OI, detailed cellular and molecular studies of heart and vascular tissues in animal models are needed. | 5 | 4 | 2 | 0 | 0 |
| To determine the prevalence and types of cardiac disorders in people with OI, cardiac and genetic evaluations of large numbers of the OI population (selected in an unbiased manner) are needed. | 10 | 1 | 0 | 0 | 0 |
| To better understand the progression of valvular and structural heart abnormalities in people with OI, longitudinal studies of cardiac function and outcomes are needed. | 10 | 1 | 0 | 0 | 0 |
