# Supplementary material for: Cardiovascular disease in adults with osteogenesis imperfecta: clinical characteristics, care recommendations, and research priorities identified using a modified Delphi technique
Source: J Bone Miner Res. 2024 Dec 12;40(2):211–21. doi: 10.1093/jbmr/zjae197 (PMC11789389; doi:10.1093/jbmr/zjae197)
Supplement: Supplement_table_4_Second_round_of_votes_zjae197 [file supplement_table_4_second_round_of_votes_zjae197.docx]

**Supplement table 4. Results of the second round of voting**

| **Round 2** |  |  |  |  |  |
| --- | --- | --- | --- | --- | --- |
| **Statement** | **Strongly Agree** | **Agree** | **Neutral** | **Disagree** | **Strongly Disagree** |
| Cardiovascular disease is a major cause of death in people with OI. | 4 | 5 | 2 | 0 | 0 |
| Current data suggest that death, on average, occurs earlier in life for people with OI as compared to the general population. | 6 | 4 | 1 | 0 | 0 |
| There is insufficient evidence to confirm that OI type and/or genotype are correlated with risk for and severity of cardiovascular disease. | 5 | 6 | 0 | 0 | 0 |
| People with OI may have myocardial structural and biomechanical abnormalities that may affect cardiac function; however the clinical implications of those abnormalities are not well understood. | 5 | 6 | 0 | 0 | 0 |
| A clinical diagnosis of heart failure and diastolic dysfunction appear to be more common in people with OI as compared to the general population. | 3 | 8 | 0 | 0 | 0 |
| Cardiac valve disease, particularly mitral and/or aortic valve insufficiency, appears to be common in people with OI, however, the clinical implications are not well understood. | 8 | 3 | 0 | 0 | 0 |
| Cardiac valve abnormalities in people with OI appear to be associated with abnormal extracellular matrix structure, myxomatous changes, and/or cystic medial degeneration. | 2 | 9 | 0 | 0 | 0 |
| Aortic root dilation appears to be more common in people with OI, however the clinical implications of such dilation are not well understood. | 5 | 5 | 1 | 0 | 0 |
| Vascular aneurysms and dissections have been reported in people with OI, however, the overall prevalence of aneurysms and risk for dissections are not well understood. | 7 | 4 | 0 | 0 | 0 |
| The prevalence of abnormal cardiovascular imaging findings is high in people with OI, however the clinical consequences of such findings are not well understood. | 7 | 3 | 1 | 0 | 0 |
| Biological plausibility of vascular disease in OI is supported by data from animal models which demonstrate histological abnormalities, as well as gross structural functional derangements in cv tissues, however, they are not well understood. | 2 | 8 | 0 | 1 | 0 |
| Baseline and periodic clinical cardiac evaluations are appropriate in all adults with OI. If abnormalities are identified, referral to a cardiovascular specialist should be considered. | 5 | 5 | 0 | 1 | 0 |
| Automated blood pressure cuffs can be used in people with OI. However, caution should be exercised in people with severe OI and/or skeletal deformity. | 4 | 6 | 0 | 1 | 0 |
| Techniques for echocardiography evaluation should be adapted to the individual's body shape and size. | 7 | 4 | 0 | 0 | 0 |
| Although the literature is limited to case reports and small case series, aortic valve and mitral valve repairs/replacements can be successful in people with OI. | 4 | 7 | 0 | 0 | 0 |
| Although the literature is limited to case reports and small case series, cardiac surgery has been reported to have a greater risk of complications in people with OI. Tissue fragility, bleeding, and poor wound healing may be more likely. | 4 | 7 | 0 | 0 | 0 |
| When people with OI undergo cardiac surgery, the following should be considered to optimize surgical outcomes & minimize complications: a. When clinically indicated, cervical spine imaging should be performed. | 5 | 4 | 1 | 1 | 0 |
| 17b. Surgery should be well-planned preoperatively by a multidisciplinary team, and preferably conducted at a center with expertise in OI. | 6 | 5 | 0 | 0 | 0 |
| 17c. The patient should be carefully positioned on the operating table with padding and support of the neck and extremities. | 7 | 4 | 0 | 0 | 0 |
| 17d. Due to the risk of surgical bleeding complications, drugs and equipment to ensure hemostasis should be available in the OR. | 5 | 6 | 0 | 0 | 0 |
| The current evidence related to cardiovascular disease in osteogenesis imperfecta is primarily limited to case reports, small case series and cross-sectional studies, which introduces publication and selection bias. | 9 | 2 | 0 | 0 | 0 |
| To better understand the nature of cardiovascular abnormalities in people with OI, detailed cellular and molecular studies in preclinical models and human tissues are needed. | 9 | 2 | 0 | 0 | 0 |
| To determine the prevalence, types & outcomes of cardiovascular disorders in people with OI, cardiovascular & genetic evaluations of large cohorts of the OI population worldwide (recruited in an unbiased manner) are needed with consideration of controls. | 7 | 4 | 0 | 0 | 0 |
| To better understand the progression of cardiovascular abnormalities in people with OI, longitudinal studies are needed to evaluate cardiovascular function and outcomes across the lifespan and by sex. | 8 | 3 | 0 | 0 | 0 |
| Future research will only be successful if conducted in a patient-centered way, with active participation and input from people with OI and other stakeholders. | 7 | 3 | 1 | 0 | 0 |
| Future endeavors should aim to develop practice resources to support healthcare providers and people with OI for appropriate evaluation and treatment of cardiovascular disease. | 6 | 5 | 0 | 0 | 0 |
