# Supplementary material for: Cardiovascular disease in adults with osteogenesis imperfecta: clinical characteristics, care recommendations, and research priorities identified using a modified Delphi technique
Source: J Bone Miner Res. 2024 Dec 12;40(2):211–21. doi: 10.1093/jbmr/zjae197 (PMC11789389; doi:10.1093/jbmr/zjae197)
Supplement: Supplement_table_5_Final_round_of_votes_zjae197 [file supplement_table_5_final_round_of_votes_zjae197.docx]

**Supplement table 5 Results of the final round of voting**

| **Manuscript Statements** |  |  |  |  |  |
| --- | --- | --- | --- | --- | --- |
| **Statement** | **Strongly Agree** | **Agree** | **Neutral** | **Disagree** | **Strongly Disagree** |
| As in the general population, cardiovascular disease is a major cause of death in people with OI. | 5 | 4 | 1 | 0 | 0 |
| The biological plausibility of CVD in OI is supported by data from animal models which demonstrate histological, structural, and functional abnormalities in cardiovascular tissues. | 6 | 4 | 0 | 0 | 0 |
| There is insufficient evidence to confirm that OI type and/or genotype are correlated with risk for and severity of cardiovascular disease. | 5 | 5 | 0 | 0 | 0 |
| Myocardial structural and biomechanical abnormalities that affect cardiac function have been reported in people with OI; however, the clinical implications of those abnormalities are not well understood. | 7 | 3 | 0 | 0 | 0 |
| Shortness of breath as a symptom is more common in people with OI than in the rest of the population. It is not clear whether this is due to cardiovascular, pulmonary, or other causes. | 8 | 2 | 0 | 0 | 0 |
| Cardiac valvular abnormalities, particularly mitral and/or aortic valve regurgitation, appears to be common in people with OI, however, the clinical implications are not well understood. | 5 | 4 | 0 | 0 | 0 |
| Aortic root dilation appears to be more common in people with OI, however the clinical implications of this dilation are not well understood. | 6 | 4 | 0 | 0 | 0 |
| Vascular aneurysms and dissections have been reported in people with OI, however, the overall prevalence of aneurysms is unknown and the risk for dissections is not well understood. | 9 | 1 | 0 | 0 | 0 |
| Baseline and periodic clinical cardiovascular evaluations are appropriate in all adults with OI. If abnormalities are identified, referral to a cardiovascular specialist should be considered. | 7 | 3 | 0 | 0 | 0 |
| Automated blood pressure cuffs can be used in people with OI. However, caution should be exercised in examining people with severe OI and/or skeletal deformity. | 4 | 5 | 1 | 0 | 0 |
| Techniques for echocardiographic evaluation of adults with OI should be adapted to the individual's body shape and size. | 8 | 2 | 0 | 0 | 0 |
| Based on limited evidence from case reports, aortic valve and mitral valve surgeries can be successful in people with OI. | 4 | 5 | 1 | 0 | 0 |
| Although the literature is limited to case reports and small case series, cardiac surgery has been reported to have a greater risk of complications in people with OI. | 4 | 6 | 0 | 0 | 0 |
| When people with OI undergo cardiovascular surgery particular care should be taken to optimize surgical outcomes and minimize complications. | 8 | 1 | 0 | 0 | 0 |
| The current evidence related to cardiovascular disease in OI is primarily limited to case reports, small case series and cross-sectional studies, which introduces publication and selection bias. | 10 |  | 0 | 0 | 0 |
| To better understand the nature of cardiovascular abnormalities in people with OI, detailed cellular and molecular studies in preclinical models and human tissues are needed. | 7 | 3 | 0 | 0 | 0 |
| To determine the prevalence, types and outcomes of cardiovascular disease in people with OI, cardiovascular and genetic evaluations of large cohorts of the OI population worldwide (recruited in an unbiased manner) are needed, with selection of appropriate controls. In addition, to better understand the progression of cardiovascular abnormalities in people with OI, longitudinal studies are needed to evaluate cardiovascular function and outcomes across the lifespan. Potential differences due to sex and race/ethnicity should be studied. | 10 | 0 | 0 | 0 | 0 |
| Future research will be strengthened if conducted in a patient-centered way, with active participation and input from people with OI and other stakeholders. | 8 | 2 | 0 | 0 | 0 |
| Future endeavors should develop resources for healthcare providers and people with OI that would facilitate appropriate evaluation and treatment of cardiovascular disease. | 8 | 2 | 0 | 0 | 0 |

*Ten out of the 11 who voted in rounds 1 and 2 participated in this vote.*
