# Supplementary material for: Cardiovascular disease in adults with osteogenesis imperfecta: clinical characteristics, care recommendations, and research priorities identified using a modified Delphi technique
Source: J Bone Miner Res. 2024 Dec 12;40(2):211–21. doi: 10.1093/jbmr/zjae197 (PMC11789389; doi:10.1093/jbmr/zjae197)
Supplement: Supplement_1_Methods_zjae197 [file supplement_1_methods_zjae197.docx]

**Supplement 1 – Detailed methods**

Development of the project.

The project arose from discussions among the members of the Adult OI Working Group, a team of clinician investigators from the U.S. and Europe that is interested in synthesizing available knowledge about OI in adults to advance public and professional understanding of the disorder, and to identify critical knowledge gaps and research opportunities. By consensus, CVD in OI was considered to be an important issue, and we knew of no existing guidelines for its evaluation or care. Whereas the group initially chose to use a modified Delphi-based approach to build consensus, the final approach has elements of both the group Delphi and nominal group techniques^138^.

The Delphi panel

Participants in the Delphi process are listed in Supplemental Table 1. Because of the members’ diverse and complementary expertise in OI, the core panel of experts undertaking the project included the 9 members of the Adult OI Working Group. The group also selected three additional clinician investigators; two cardiologists and one pulmonologist. Finally, a genetic counselor interested in OI was included. An experienced facilitator was recruited to aid in developing the process, and in discussions and consensus development. Support for the project and administrative assistance was provided by the Osteogenesis Imperfecta Foundation, a national health organization dedicated to helping people cope with the problems associated with OI. The investigators devoted their time without project-based remuneration.

Data sources and search strategy

A literature search was conducted to generate a comprehensive list of published articles about CVD in OI. An initial search of PubMed was performed in December 2023 and retrieved studies published between 1960 and 2023. That search was reviewed and updated in December 2023. The literature search process is described in the Supplemental Methods and summarized in Supplemental Figure 1. The search terms used are listed in Supplemental Table 2.

Whereas the project primarily reviewed articles involving cardiovascular disease in adults with OI, similar articles about pediatric OI and cardiovascular studies in animal models were also included for their relevance in interpreting adult disease. Articles screened by four individuals (analysts from OHSU, Hospital for Special Surgery and the Osteogenesis Imperfecta Foundation) using a prespecified search protocol. The included articles were restricted to reports published in English and involving humans and mouse models. Abstracts were reviewed for relevant reports, and full articles were reviewed when necessary to determine their relevance. To some extent, the several PubMed search terms that were used yielded overlapping results, and duplicated articles were included only once. Reference lists from identified articles were also examined by all members of the panel to identify potentially relevant articles missing from the original search.

Overall, 4393 citations were screened, and 165 were deemed to meet search criteria. This number is similar to what other reviews have identified using similar, but not identical search strategies^9,10^. Of these, full text versions were available for 137 citations. The article list was collated in a spread sheet (with article titles, links to PubMed citations, PMID codes and dates published) and distributed to panel members. Additionally, copies of each publication were made available in a secure, online shared dataset. Each panel member reviewed all of the literature in December 2023-January 2024.

Consensus development

A 3-day meeting with rounds of discussion and initial consensus development was held in January 2024. Seven panel members participated in-person and 4 members participated by webconference. Prior to the meeting, a set of 26 draft statements was prepared by 2 members of the panel (EO, CR) and distributed to panel members. The statements were used as guidestones to summarize the key elements of the literature, provide clinical guidance based on available data, and identify critical areas of research needed to address gaps. Statements were grouped into three categories: summaries of the knowledge about key cardiovascular issues in adults with OI, clinical recommendations, and research priorities. Prior to the meeting, an anonymous vote (strongly agree, agree, neutral, disagree, strongly disagree) was taken concerning each statement in sequence. During the meeting, each statement was critically discussed and modified by the group. At the conclusion of that process, the statements were all again considered, discussed, and modified, and a concluding anonymous vote was taken. AT the conclusion of the initial meeting, the members of the group divided the statements among themselves for drafting text describing the background and rationale that led to the development of each of the statements. In addition, a brief clinical guidance or recommendation was written. After the background and recommendations were written, assembled edited, a final anonymous vote was taken. The results of the 3 stages of voting are provided in Supplemental Tables 3-5.

Manuscript composition and final consensus

Members of the consensus development group wrote text describing the literature underlying the background for each statement. In the process of refining one statement, 2 additional search terms were employed yielding 15 additional articles (Supplemental Table 2). At that point, the manuscript was provided to each member of the panel for review and editing. Another meeting of the group was convened by video during which the manuscript, and each statement, was discussed, and minor modifications were made in the statements and their background and rationale sections. A final vote was taken on each of the statements. The initial draft manuscript text was written and assembled by April 1, 2024, and the final manuscript was approved for submission by July 1, 2024. Our approach was designed to adhere to the guidelines proposed in the Accurate Consensus Reporting Document (ACCORD) for the description of consensus methods in biomedicine^138^.
