# Supplementary material for: Cardiovascular disease in adults with osteogenesis imperfecta: clinical characteristics, care recommendations, and research priorities identified using a modified Delphi technique
Source: J Bone Miner Res. 2024 Dec 12;40(2):211–21. doi: 10.1093/jbmr/zjae197 (PMC11789389; doi:10.1093/jbmr/zjae197)
Supplement: Supplement_2_Full_list_of_references_zjae197 [file supplement_2_full_list_of_references_zjae197.docx]

**Supplement 2 - Full list of references**

1. Marini JC, Forlino A, Bächinger HP, et al. Osteogenesis imperfecta. *Nat Rev Dis Primers*. Aug 18 2017;3:17052. doi:10.1038/nrdp.2017.52

2. Yu H, Li C, Wu H, et al. Pathogenic mechanisms of osteogenesis imperfecta, evidence for classification. *Orphanet J Rare Dis*. Aug 9 2023;18(1):234. doi:10.1186/s13023-023-02849-5

3. Marom R, Rabenhorst BM, Morello R. Osteogenesis imperfecta: an update on clinical features and therapies. *Eur J Endocrinol*. Oct 2020;183(4):R95-r106. doi:10.1530/eje-20-0299

4. Kaliaperumal C, Walsh T, Balasubramanian C, Wyse G, Fanning N, Kaar G. Osteogenesis imperfecta presenting as aneurysmal subarachnoid haemorrhage in a 53-year-old man. *BMJ Case Rep*. Nov 21 2011;2011doi:10.1136/bcr.10.2011.4910

5. Robinson TF, Cohen-Gould L, Factor SM, Eghbali M, Blumenfeld OO. Structure and function of connective tissue in cardiac muscle: collagen types I and III in endomysial struts and pericellular fibers. *Scanning Microsc*. Jun 1988;2(2):1005-15.

6. Cole WG, Chan D, Hickey AJ, Wilcken DE. Collagen composition of normal and myxomatous human mitral heart valves. *Biochem J*. Apr 15 1984;219(2):451-60. doi:10.1042/bj2190451

7. Eghbali M, Weber KT. Collagen and the myocardium: fibrillar structure, biosynthesis and degradation in relation to hypertrophy and its regression. *Mol Cell Biochem*. Jul 17 1990;96(1):1-14. doi:10.1007/bf00228448

8. Hinton RB, Yutzey KE. Heart valve structure and function in development and disease. *Annu Rev Physiol*. 2011;73:29-46. doi:10.1146/annurev-physiol-012110-142145

9. Ashournia H, Johansen FT, Folkestad L, Diederichsen AC, Brixen K. Heart disease in patients with osteogenesis imperfecta - A systematic review. *Int J Cardiol*. Oct 1 2015;196:149-57. doi:10.1016/j.ijcard.2015.06.001

10. Verdonk SJE, Storoni S, Micha D, et al. Is Osteogenesis Imperfecta Associated with Cardiovascular Abnormalities? A Systematic Review of the Literature. *Calcif Tissue Int*. Mar 2024;114(3):210-221. doi:10.1007/s00223-023-01171-3

11. Jones J, Hunter D. Consensus methods for medical and health services research. *BMJ*. Aug 5 1995;311(7001):376-80. doi:10.1136/bmj.311.7001.376

12. Folkestad L, Hald JD, Canudas-Romo V, et al. Mortality and Causes of Death in Patients With Osteogenesis Imperfecta: A Register-Based Nationwide Cohort Study. *J Bone Miner Res*. Dec 2016;31(12):2159-2166. doi:10.1002/jbmr.2895

13. Folkestad L, Hald JD, Gram J, et al. Cardiovascular disease in patients with osteogenesis imperfecta - a nationwide, register-based cohort study. *Int J Cardiol*. Dec 15 2016;225:250-257. doi:10.1016/j.ijcard.2016.09.107

14. McAllion SJ, Paterson CR. Causes of death in osteogenesis imperfecta. *J Clin Pathol*. Aug 1996;49(8):627-30. doi:10.1136/jcp.49.8.627

15. Caulfield JB, Borg TK. The collagen network of the heart. *Lab Invest*. Mar 1979;40(3):364-72.

16. Robinson TF, Geraci MA, Sonnenblick EH, Factor SM. Coiled perimysial fibers of papillary muscle in rat heart: morphology, distribution, and changes in configuration. *Circ Res*. Sep 1988;63(3):577-92. doi:10.1161/01.res.63.3.577

17. Rodriguez KJ, Piechura LM, Porras AM, Masters KS. Manipulation of valve composition to elucidate the role of collagen in aortic valve calcification. *BMC Cardiovasc Disord*. Mar 1 2014;14:29. doi:10.1186/1471-2261-14-29

18. Taylor PM. Biological matrices and bionanotechnology. *Philos Trans R Soc Lond B Biol Sci*. Aug 29 2007;362(1484):1313-20. doi:10.1098/rstb.2007.2117

19. Shekhonin BV, Domogatsky SP, Muzykantov VR, Idelson GL, Rukosuev VS. Distribution of type I, III, IV and V collagen in normal and atherosclerotic human arterial wall: immunomorphological characteristics. *Coll Relat Res*. Sep 1985;5(4):355-68. doi:10.1016/s0174-173x(85)80024-8

20. Jana S, Hu M, Shen M, Kassiri Z. Extracellular matrix, regional heterogeneity of the aorta, and aortic aneurysm. *Exp Mol Med*. Dec 19 2019;51(12):1-15. doi:10.1038/s12276-019-0286-3

21. Lisse TS, Thiele F, Fuchs H, et al. ER stress-mediated apoptosis in a new mouse model of osteogenesis imperfecta. *PLoS Genet*. Feb 2008;4(2):e7. doi:10.1371/journal.pgen.0040007

22. Thiele F, Cohrs CM, Flor A, et al. Cardiopulmonary dysfunction in the Osteogenesis imperfecta mouse model Aga2 and human patients are caused by bone-independent mechanisms. *Hum Mol Genet*. Aug 15 2012;21(16):3535-45. doi:10.1093/hmg/dds183

23. Chen F, Guo R, Itoh S, et al. First mouse model for combined osteogenesis imperfecta and Ehlers-Danlos syndrome. *J Bone Miner Res*. Jun 2014;29(6):1412-23. doi:10.1002/jbmr.2177

24. Weis SM, Emery JL, Becker KD, McBride DJ, Jr., Omens JH, McCulloch AD. Myocardial mechanics and collagen structure in the osteogenesis imperfecta murine (oim). *Circ Res*. Oct 13 2000;87(8):663-9. doi:10.1161/01.res.87.8.663

25. Pfeiffer BJ, Franklin CL, Hsieh FH, Bank RA, Phillips CL. Alpha 2(I) collagen deficient oim mice have altered biomechanical integrity, collagen content, and collagen crosslinking of their thoracic aorta. *Matrix Biol*. Oct 2005;24(7):451-8. doi:10.1016/j.matbio.2005.07.001

26. Thatcher K, Mattern CR, Chaparro D, et al. Temporal Progression of Aortic Valve Pathogenesis in a Mouse Model of Osteogenesis Imperfecta. *J Cardiovasc Dev Dis*. Aug 20 2023;10(8)doi:10.3390/jcdd10080355

27. Ben Amor IM, Glorieux FH, Rauch F. Genotype-phenotype correlations in autosomal dominant osteogenesis imperfecta. *J Osteoporos*. 2011;2011:540178. doi:10.4061/2011/540178

28. Maioli M, Gnoli M, Boarini M, et al. Genotype-phenotype correlation study in 364 osteogenesis imperfecta Italian patients. *Eur J Hum Genet*. Jul 2019;27(7):1090-1100. doi:10.1038/s41431-019-0373-x

29. Evin F, Aydın D, Levent E, Özen S, Darcan Ş, Gökşen D. A case-control study of early-stage radiological markers of endothelial dysfunction and cardiovascular findings in patients with osteogenesis imperfecta: genotype-phenotype correlations. *J Pediatr Endocrinol Metab*. Dec 15 2023;36(12):1161-1168. doi:10.1515/jpem-2023-0215

30. Vandersteen AM, Lund AM, Ferguson DJ, et al. Four patients with Sillence type I osteogenesis imperfecta and mild bone fragility, complicated by left ventricular cardiac valvular disease and cardiac tissue fragility caused by type I collagen mutations. *Am J Med Genet A*. Feb 2014;164a(2):386-91. doi:10.1002/ajmg.a.36285

31. Zhao D, Liu Y, Liu J, et al. Cardiovascular abnormalities and its correlation with genotypes of children with osteogenesis imperfecta. *Front Endocrinol (Lausanne)*. 2022;13:1004946. doi:10.3389/fendo.2022.1004946

32. Hortop J, Tsipouras P, Hanley JA, Maron BJ, Shapiro JR. Cardiovascular involvement in osteogenesis imperfecta. *Circulation*. Jan 1986;73(1):54-61. doi:10.1161/01.cir.73.1.54

33. Migliaccio S, Barbaro G, Fornari R, et al. Impairment of diastolic function in adult patients affected by osteogenesis imperfecta clinically asymptomatic for cardiac disease: casuality or causality? *Int J Cardiol*. Jan 9 2009;131(2):200-3. doi:10.1016/j.ijcard.2007.10.051

34. Lamanna A, Fayers T, Clarke S, Parsonage W. Valvular and aortic diseases in osteogenesis imperfecta. *Heart Lung Circ*. Oct 2013;22(10):801-10. doi:10.1016/j.hlc.2013.05.640

35. Radunovic Z, Wekre LL, Steine K. Right ventricular and pulmonary arterial dimensions in adults with osteogenesis imperfecta. *Am J Cardiol*. Jun 15 2012;109(12):1807-13. doi:10.1016/j.amjcard.2012.01.402

36. Radunovic Z, Wekre LL, Diep LM, Steine K. Cardiovascular abnormalities in adults with osteogenesis imperfecta. *Am Heart J*. Mar 2011;161(3):523-9. doi:10.1016/j.ahj.2010.11.006

37. Hernández Jiménez V, Saavedra Falero J, Alberca Vela MT, Mata Caballero R, Rosado Sierra JA, Pavón de Paz I. Structural and functional changes in the heart of adult patients with osteogenesis imperfecta: Case-control study. *Med Clin (Barc)*. Nov 21 2018;151(10):397-399. Cambios estructurales y funcionales en el corazón de pacientes adultos con osteogénesis imperfecta: estudio de casos y controles. doi:10.1016/j.medcli.2018.02.010

38. Radunovic Z, Steine K. Prevalence of Cardiovascular Disease and Cardiac Symptoms: Left and Right Ventricular Function in Adults With Osteogenesis Imperfecta. *Can J Cardiol*. Nov 2015;31(11):1386-92. doi:10.1016/j.cjca.2015.04.016

39. RA S. Pulmonary Function in Osteogenesis Imperfecta. In: J S, ed. *Osteogenesis Imperfecta: A Translational Approach to Brittle Bone Disease*. Academic Press/Elsevier; 2014:335-342.

40. Tam A, Chen S, Schauer E, et al. A multicenter study to evaluate pulmonary function in osteogenesis imperfecta. *Clin Genet*. Dec 2018;94(6):502-511. doi:10.1111/cge.13440

41. Gochuico BR, Hossain M, Talvacchio SK, et al. Pulmonary function and structure abnormalities in children and young adults with osteogenesis imperfecta point to intrinsic and extrinsic lung abnormalities. *J Med Genet*. Nov 2023;60(11):1067-1075. doi:10.1136/jmg-2022-109009

42. Chaney H, Mekking D, De Bakker D, et al. Key4OI Recommendations for Lung Function Guidance in Osteogenesis Imperfecta: Based on an Internationally Performed Comprehensive International Consortium for Health Outcomes Measurement Procedure. *Chest*. May 2023;163(5):1201-1213. doi:10.1016/j.chest.2022.12.047

43. Westerheim I, Hart T, van Welzenis T, et al. The IMPACT survey: a mixed methods study to understand the experience of children, adolescents and adults with osteogenesis imperfecta and their caregivers. *Orphanet J Rare Dis*. Mar 21 2024;19(1):128. doi:10.1186/s13023-024-03126-9

44. Yancy CW, Jessup M, Bozkurt B, et al. 2013 ACCF/AHA guideline for the management of heart failure: a report of the American College of Cardiology Foundation/American Heart Association Task Force on practice guidelines. *Circulation*. Oct 15 2013;128(16):e240-327. doi:10.1161/CIR.0b013e31829e8776

45. Global, regional, and national burden of congenital heart disease, 1990-2017: a systematic analysis for the Global Burden of Disease Study 2017. *Lancet Child Adolesc Health*. Mar 2020;4(3):185-200. doi:10.1016/s2352-4642(19)30402-x

46. Singh JP, Evans JC, Levy D, et al. Prevalence and clinical determinants of mitral, tricuspid, and aortic regurgitation (the Framingham Heart Study). *Am J Cardiol*. Mar 15 1999;83(6):897-902. doi:10.1016/s0002-9149(98)01064-9

47. Kalath S, Tsipouras P, Silver FH. Increased aortic root stiffness associated with osteogenesis imperfecta. *Ann Biomed Eng*. 1987;15(1):91-9. doi:10.1007/bf02364170

48. Matouk CC, Hanbidge A, Mandell DM, Terbrugge KG, Agid R. Osteogenesis imperfecta, multiple intra-abdominal arterial dissections and a ruptured dissecting-type intracranial aneurysm. *Interv Neuroradiol*. Sep 2011;17(3):371-5. doi:10.1177/159101991101700315

49. Matsushiro M, Harada D, Ueyama K, et al. Intracranial aneurysm as a possible complication of osteogenesis imperfecta: a case series and literature review. *Endocr J*. Jul 28 2023;70(7):697-702. doi:10.1507/endocrj.EJ22-0620

50. Tang X, Jian J, Luo Y, Fan H, Liu P, Chen Y. Spontaneous extracranial arterial dissections in a case of patient with osteogenesis imperfecta. *Int J Neurosci*. Mar 2021;131(3):312-316. doi:10.1080/00207454.2020.1739674

51. Sardana V, Kamble S, Sharma SK, Maheshwari D, Bhushan B. Mirror Aneurysm with Right Frontal ICH in a Patient with Osteogenesis Imperfecta. *J Assoc Physicians India*. Aug 2017;65(8):103-105.

52. Kolukısa M, Gökçal E, Gürsoy AE, Deniz Ç, Aralaşmak A, Asil T. Multiple Spontaneous Intracranial-Extracranial Arterial Dissections in a Patient with Osteogenesis Imperfecta. *Case Rep Neurol Med*. 2017;2017:8520961. doi:10.1155/2017/8520961

53. Mansfield K, Rahme R. Dissecting Aneurysm of the Recurrent Artery of Heubner in a Patient With Osteogenesis Imperfecta. *Can J Neurol Sci*. Nov 2015;42(6):461-5. doi:10.1017/cjn.2015.295

54. Hirohata T, Miyawaki S, Mizutani A, et al. Subarachnoid hemorrhage secondary to a ruptured middle cerebral aneurysm in a patient with osteogenesis imperfecta: a case report. *BMC Neurol*. Jul 23 2014;14:150. doi:10.1186/1471-2377-14-150

55. Havlik DM, Nashelsky MB. Ruptured cerebral artery aneurysm and bacterial meningitis in a man with osteogenesis imperfecta. *Am J Forensic Med Pathol*. Jun 2006;27(2):117-20. doi:10.1097/01.paf.0000203150.95087.b6

56. Kato Y, Nagoya H, Abe T, et al. Progressive Bilateral Vertebral Artery Dissection in a Case of Osteogenesis Imperfecta. *J Stroke Cerebrovasc Dis*. Mar 2017;26(3):e43-e46. doi:10.1016/j.jstrokecerebrovasdis.2016.12.012

57. Labedi A, Hoepner R, Lukas C, Meves SH, Krogias C. Multiple dysgenesis of brain supplying arteries in a patient with osteogenesis imperfecta. *Neurol Sci*. Jul 2014;35(7):1153-4. doi:10.1007/s10072-014-1710-z

58. Gaberel T, Rochey A, di Palma C, Lucas F, Touze E, Emery E. Ruptured intracranial aneurysm in patients with osteogenesis imperfecta: 2 familial cases and a systematic review of the literature. *Neurochirurgie*. Dec 2016;62(6):317-320. doi:10.1016/j.neuchi.2016.07.004

59. Takeda R, Yamaguchi T, Hayashi S, et al. Clinical and molecular features of patients with COL1-related disorders: Implications for the wider spectrum and the risk of vascular complications. *Am J Med Genet A*. Sep 2022;188(9):2560-2575. doi:10.1002/ajmg.a.62887

60. Vaish AK, Kumar N, Jain N, Agarwal A. Osteogenesis imperfecta with right renal artery occlusion. *BMJ Case Rep*. Sep 7 2012;2012doi:10.1136/bcr-2012-006536

61. Hajsadeghi S, Jafarian Kerman SR, Pouraliakbar H, Mohammadi R. A huge coronary artery aneurysm in osteogenesis imperfecta: a case report. *Acta Med Iran*. 2012;50(11):785-8.

62. Folkestad L. Mortality and morbidity in patients with osteogenesis imperfecta in Denmark. *Dan Med J*. Apr 2018;65(4)

63. Isotalo PA, Guindi MM, Bedard P, Brais MP, Veinot JP. Aortic dissection: a rare complication of osteogenesis imperfecta. *Can J Cardiol*. Oct 1999;15(10):1139-42.

64. Acar J, Breil B, Lavabre J, et al. [Osteogenesis imperfecta and aortic incompetence. One case with pathological findings. Review of the literature (author's transl)]. *Ann Med Interne (Paris)*. 1980;131(8):514-8. Ostéogenèse imparfaite et insuffisance aortique. Une observation avec documents anatomiques. Revue de la littérature.

65. Dimitrakakis G, Challoumas D, von Oppell UO. What type of valve is most appropriate for osteogenesis imperfecta patients? *Interact Cardiovasc Thorac Surg*. Sep 2014;19(3):499-504. doi:10.1093/icvts/ivu152

66. Wong RS, Follis FM, Shively BK, Wernly JA. Osteogenesis imperfecta and cardiovascular diseases. *Ann Thorac Surg*. Nov 1995;60(5):1439-43. doi:10.1016/0003-4975(95)00706-q

67. Ju H, Dixon IM. Extracellular matrix and cardiovascular diseases. *Can J Cardiol*. Dec 1996;12(12):1259-67.

68. Millington-Sanders C, Meir A, Lawrence L, Stolinski C. Structure of chordae tendineae in the left ventricle of the human heart. *J Anat*. May 1998;192 ( Pt 4)(Pt 4):573-81. doi:10.1046/j.1469-7580.1998.19240573.x

69. Sullivan BT, Margalit A, Garg VS, Njoku DB, Sponseller PD. Incidence of Fractures From Perioperative Blood Pressure Cuff Use, Tourniquet Use, and Patient Positioning in Osteogenesis Imperfecta. *J Pediatr Orthop*. Jan 2019;39(1):e68-e70. doi:10.1097/bpo.0000000000001105

70. Rothschild L, Goeller JK, Voronov P, Barabanova A, Smith P. Anesthesia in children with osteogenesis imperfecta: Retrospective chart review of 83 patients and 205 anesthetics over 7 years. *Paediatr Anaesth*. Nov 2018;28(11):1050-1058. doi:10.1111/pan.13504

71. Silbiger JJ, Parikh A. Pectus excavatum: echocardiographic, pathophysiologic, and surgical insights. *Echocardiography*. Aug 2016;33(8):1239-44. doi:10.1111/echo.13269

72. Isselbacher EM, Preventza O, Hamilton Black J, 3rd, et al. 2022 ACC/AHA guideline for the diagnosis and management of aortic disease: A report of the American Heart Association/American College of Cardiology Joint Committee on Clinical Practice Guidelines. *J Thorac Cardiovasc Surg*. Nov 2023;166(5):e182-e331. doi:10.1016/j.jtcvs.2023.04.023

73. Lopez L, Colan S, Stylianou M, et al. Relationship of Echocardiographic Z Scores Adjusted for Body Surface Area to Age, Sex, Race, and Ethnicity: The Pediatric Heart Network Normal Echocardiogram Database. *Circ Cardiovasc Imaging*. Nov 2017;10(11)doi:10.1161/circimaging.117.006979

74. Lang RM, Badano LP, Mor-Avi V, et al. Recommendations for cardiac chamber quantification by echocardiography in adults: an update from the American Society of Echocardiography and the European Association of Cardiovascular Imaging. *J Am Soc Echocardiogr*. Jan 2015;28(1):1-39.e14. doi:10.1016/j.echo.2014.10.003

75. Buchkremer F, Segerer S. Body Surface Area, Creatinine Excretion Rate, and Total Body Water: Reference Data for Adults in the United States. *Kidney Med*. Mar-Apr 2021;3(2):312-313. doi:10.1016/j.xkme.2020.10.009

76. Aoki T, Kuraoka S, Ohtani S, Kuroda Y. Aortic valve replacement in a woman with osteogenesis imperfecta. *Ann Thorac Cardiovasc Surg*. Feb 2002;8(1):51-3.

77. Yao JV, Thakkar HV, Sethwala A, Peters S, Winship I. Left Main Spontaneous Coronary Artery Dissection in a Patient With Osteogenesis Imperfecta: Use of Multimodal Imaging. *Heart Lung Circ*. Nov 2023;32(11):e81-e82. doi:10.1016/j.hlc.2023.09.015

78. Suzuki K, Sezai A, Unosawa S, Hao H, Tanaka M. Aortic valve replacement for aortic regurgitation associated with osteogenesis imperfecta. *Cardiovasc Pathol*. Sep-Oct 2018;36:11-14. doi:10.1016/j.carpath.2018.05.004

79. Güllü A, Senay S, Ozkan B, Kocyigit M, Alhan C. Aortic valve reconstruction with autologous pericardium in a patient with osteogenesis imperfecta. *J Surg Case Rep*. Oct 2018;2018(10):rjy251. doi:10.1093/jscr/rjy251

80. Minol JP, Zeus T, Blehm A, Veulemans V. TAVI as Therapy of Choice for Aortic Valve Disease in Osteogenesis Imperfecta. *J Heart Valve Dis*. Jan 2018;27(1):104-106.

81. Melly L, Dincq AS, Hanet C, Rondelet B. Case report: osteogenesis imperfecta, internal mammary artery graft & nitinol clips. *J Cardiothorac Surg*. Dec 19 2017;12(1):117. doi:10.1186/s13019-017-0685-2

82. Joshi P, Thakur S, Finn C, Sadlier P. Sternal-Sparing Aortic Valve Replacement in a Patient with Osteogenesis Imperfecta: A Case Report. *J Heart Valve Dis*. Nov 2017;26(6):744-746.

83. Tagliasacchi I, Martinelli L, Bardaro L, Chierchia S. Minimally invasive mitral valve repair in osteogenesis imperfecta. *Interact Cardiovasc Thorac Surg*. Oct 1 2017;25(4):665-666. doi:10.1093/icvts/ivx164

84. Itoda Y, Nawata K, Yamauchi H, Kinoshita O, Kimura M, Ono M. Central aortic valve closure successfully treated aortic insufficiency of the patient with Jarvik 2000 continuous flow left ventricular assist device: a case report. *J Artif Organs*. Mar 2017;20(1):99-101. doi:10.1007/s10047-016-0929-2

85. Sumi M, Ariyoshi T, Matsukuma S, et al. Surgical technique of double valve replacement in a patient with osteogenesis imperfecta. *Gen Thorac Cardiovasc Surg*. Apr 2016;64(4):220-3. doi:10.1007/s11748-014-0433-7

86. Santoro ML, Kogika MM, Hagiwara MK, Mirandola RM, Castelar IL. Decreased erythrocyte osmotic fragility during canine leptospirosis. *Rev Inst Med Trop Sao Paulo*. Jan-Feb 1994;36(1):1-5. doi:10.1590/s0036-46651994000100001

87. Concistrè G, Casali G, Della Monica PL, et al. Aortic valve replacement in a patient with ostegenesis imperfecta A case report. *Ann Ital Chir*. Nov-Dec 2014;85(6):593-5.

88. van der Kley F, Delgado V, Ajmone Marsan N, Schalij MJ. Transcatheter mitral valve repair in osteogenesis imperfecta associated mitral valve regurgitation. *Heart Lung Circ*. Aug 2014;23(8):e169-71. doi:10.1016/j.hlc.2014.03.025

89. Dimitrakakis G, Rathod J, von Oppell UO, Pericleous A, Hutchison S. Mini-sternotomy approach for aortic valve replacement in a patient with osteogenesis imperfecta. *Cardiovasc J Afr*. Oct 23 2013;24(9-10):e4-7. doi:10.5830/cvja-2013-070

90. Najib MQ, Schaff HV, Ganji J, et al. Valvular heart disease in patients with osteogenesis imperfecta. *J Card Surg*. Mar 2013;28(2):139-43. doi:10.1111/jocs.12064

91. Pfannmueller B, Borger MA, Battellini RR, Mohr FW. Mitral valve re-replacement in a patient with osteogenesis imperfecta. *Thorac Cardiovasc Surg*. Dec 2010;58(8):486-8. doi:10.1055/s-0030-1249868

92. Yamabi H, Imanaka K, Sato H, Matsuoka T. [Aortic valve replacement without blood transfusion in a patient with osteogenesis imperfecta]. *Kyobu Geka*. Mar 2010;63(3):208-11.

93. Wakiyama H, Okada Y, Kitamura A, Imai Y. Aortic root replacement with a stentless bioprosthesis in osteogenesis imperfecta. *J Heart Valve Dis*. Mar 2008;17(2):197-9.

94. Badmanaban B, Sachithanandan A, MacGowan SW. Aortic valve replacement in osteogenesis imperfecta--technical and practical considerations for a successful outcome. *J Card Surg*. Nov-Dec 2003;18(6):554-6. doi:10.1046/j.0886-0440.2003.02069.x

95. Eskola MJ, Niemelä KO, Kuusinen PR, Tarkka MR. Coronary artery dissection, combined aortic valve replacement and coronary bypass grafting in osteogenesis imperfecta. *Interact Cardiovasc Thorac Surg*. Dec 2002;1(2):83-5. doi:10.1016/s1569-9293(02)00060-9

96. Ohuchi S, Koizumi J, Kin H, et al. [Valvular heart surgery in osteogenesis imperfecta]. *Kyobu Geka*. Nov 2002;55(12):1011-3.

97. Chrysant GS, Cassivi SD, Carey CF, Sundt TM. Double valve replacement in a patient with osteogenesis imperfecta. *J Heart Valve Dis*. Sep 2002;11(5):751-4.

98. Kastrup M, von Heymann C, Hotz H, et al. Recombinant factor VIIa after aortic valve replacement in a patient with osteogenesis imperfecta. *Ann Thorac Surg*. Sep 2002;74(3):910-2. doi:10.1016/s0003-4975(02)03700-1

99. Iha K, Uehara T, Higa N, Akasaki M, Kuniyoshi Y, Koja K. Cardiac reoperation in a patient with osteogenesis imperfecta: a case report. *Ann Thorac Cardiovasc Surg*. Aug 2001;7(4):241-5.

100. Izzat MB, Wan S, Wan IY, Khaw KS, Yim AP. Ministernotomy for aortic valve replacement in a patient with osteogenesis imperfecta. *Ann Thorac Surg*. Apr 1999;67(4):1171-3. doi:10.1016/s0003-4975(99)00126-5

101. Lijoi A, Cisico S, Caputo E, Scarano F, Parodi E, Passerone GC. Left ventricular rupture after mitral valve replacement in a patient with osteogenesis imperfecta tarda. *Tex Heart Inst J*. 1999;26(4):295-7.

102. Maekawa Y, Hayashi T, Fujito T, et al. [Successful surgical treatment of aortic regurgitation due to annuloaortic ectasia and mitral regurgitation caused by tendon rupture in a case of osteogenesis imperfecta]. *J Cardiol*. 1997;29 Suppl 2:89-94.

103. Reguillo F, De La Llana R, Castañón J, et al. Osteogenesis imperfecta and coronary artery surgery. A case report. *J Cardiovasc Surg (Torino)*. Dec 1996;37(6):621-2.

104. Ichikawa H, Ishikawa S, Otaki A, et al. [Left ventricular rupture following aortic and mitral valve replacement in a patient with osteogenesis imperfecta: a case report]. *Kyobu Geka*. Apr 1996;49(4):294-6.

105. Cusimano RJ. Repeat cardiac operation in a patient with osteogenesis imperfecta. *Ann Thorac Surg*. Apr 1996;61(4):1294. doi:10.1016/0003-4975(96)81299-9

106. Almassi GH, Hughes GR, Bartlett J. Combined valve replacement and coronary bypass grafting in osteogenesis imperfecta. *Ann Thorac Surg*. Nov 1995;60(5):1395-7. doi:10.1016/0003-4975(95)00490-c

107. Tardif JC, Taylor K, Pandian NG, Schwartz S, Rastegar H. Right ventricular outflow tract and pulmonary artery obstruction by postoperative mediastinal hematoma: delineation by multiplane transesophageal echocardiography. *J Am Soc Echocardiogr*. Jul-Aug 1994;7(4):400-4. doi:10.1016/s0894-7317(14)80199-5

108. Ohteki H, Ohtsubo S, Sakurai J, Koga N, Kohchi K, Itoh T. Aortic regurgitation and aneurysm of Sinus of Valsalva associated with osteogenesis imperfecta. *Thorac Cardiovasc Surg*. Oct 1991;39(5):294-5. doi:10.1055/s-2007-1019989

109. Jeyamalar R, Hashim R, Kannan P. Aortic valve replacement in osteogenesis imperfecta tarda--a case report. *Singapore Med J*. Jun 1989;30(3):316-7.

110. Gerlach PA, Rosensweig J, Ramanathan KB. Successful aortic valve replacement in osteogenesis imperfecta: with special emphasis on peri-operative management. *Can J Cardiol*. Apr 1987;3(3):132-5.

111. Passmore JM, Walker WE, Fuentes F. Successful aortocoronary bypass in osteogenesis imperfecta. *J Am Coll Cardiol*. Apr 1987;9(4):960-3. doi:10.1016/s0735-1097(87)80255-3

112. Koentges D, van de Werf F, Stalpaert J, Goddeeris P, de Geest H. Aortic and mitral valve replacement in osteogenesis imperfecta. Report of a case. *Acta Cardiol*. 1986;41(2):147-53.

113. Jansen W, Hombach V, Osterspey A, et al. [Valve replacement for mitral in incompetence in a patient with osteogenesis imperfecta (author's transl)]. *Z Kardiol*. May 1982;71(5):357-64. Herzklappenersatzoperation bei hochgradiger Mitralinsuffizienz im Rahmen der Osteogenesis imperfecta tarda.

114. Waters DD, Clark DW, Symbas PN, Schlant RC. Aortic and mitral valve replacement in a patient with osteogenesis imperfecta. *Chest*. Sep 1977;72(3):363-4. doi:10.1378/chest.72.3.363

115. Cohen IM, Vieweg WV, Alpert JS, Kaufman JA, Hagan AD. Osteogenesis imperfecta tarda. Cardiovascular pathology. *West J Med*. Mar 1977;126(3):228-31.

116. Siggers DC. Osteogenesis imperfecta with aortic valve replacement. *Birth Defects Orig Artic Ser*. 1975;11(2):347-8.

117. Weisinger B, Glassman E, Spencer FC, Berger A. Successful aortic valve replacement for aortic regurgitation associated with osteogenesis imperfecta. *Br Heart J*. May 1975;37(5):475-7. doi:10.1136/hrt.37.5.475

118. Siggers DC. Osteogenesis imperfecta with aortic valve replacement. *Birth Defects Orig Artic Ser*. 1974;10(12):495-8.

119. Wood SJ, Thomas J, Braimbridge MV. Mitral valve disease and open heart surgery in osteogenesis imperfecta tarda. *Br Heart J*. Jan 1973;35(1):103-6. doi:10.1136/hrt.35.1.103

120. Bonita RE, Cohen IS, Berko BA. Valvular heart disease in osteogenesis imperfecta: presentation of a case and review of the literature. *Echocardiography*. Jan 2010;27(1):69-73. doi:10.1111/j.1540-8175.2009.00973.x

121. Gooijer K, Rondeel JMM, van Dijk FS, Harsevoort AGJ, Janus GJM, Franken AAM. Bleeding and bruising in Osteogenesis Imperfecta: International Society on Thrombosis and Haemostasis bleeding assessment tool and haemostasis laboratory assessment in 22 individuals. *Br J Haematol*. Nov 2019;187(4):509-517. doi:10.1111/bjh.16097

122. Hansen B, Jemec GB. The mechanical properties of skin in osteogenesis imperfecta. *Arch Dermatol*. Jul 2002;138(7):909-11. doi:10.1001/archderm.138.7.909

123. Marulanda J, Retrouvey JM, Lee B, Sutton VR, Rauch F, Briner M. Cranio-cervical abnormalities in moderate-to-severe osteogenesis imperfecta - Genotypic and phenotypic determinants. *Orthod Craniofac Res*. Apr 2024;27(2):237-243. doi:10.1111/ocr.12707

124. Cheung MS, Arponen H, Roughley P, et al. Cranial base abnormalities in osteogenesis imperfecta: phenotypic and genotypic determinants. *J Bone Miner Res*. Feb 2011;26(2):405-13. doi:10.1002/jbmr.220

125. Arponen H, Mäkitie O, Haukka J, et al. Prevalence and natural course of craniocervical junction anomalies during growth in patients with osteogenesis imperfecta. *J Bone Miner Res*. May 2012;27(5):1142-9. doi:10.1002/jbmr.1555

126. Menezes AH, Traynelis VC. Pediatric cervical kyphosis in the MRI era (1984-2008) with long-term follow up: literature review. *Childs Nerv Syst*. Feb 2022;38(2):361-377. doi:10.1007/s00381-021-05409-z

127. Pargas C, Franzone JM, Rogers KJ, et al. Cervical kyphosis: A predominant feature of patients with osteogenesis imperfecta type 5. *Bone Rep*. Dec 2020;13:100735. doi:10.1016/j.bonr.2020.100735

128. Huang J, Dinh M, Kuchle N, Zhou J. Anesthetic management for combined mitral valve replacement and aortic valve repair in a patient with osteogenesis imperfecta. *Ann Card Anaesth*. May-Aug 2011;14(2):115-8. doi:10.4103/0971-9784.81566

129. Wang H, Huang X, Wu A, Li Q. Management of anesthesia in a patient with osteogenesis imperfecta and multiple fractures: a case report and review of the literature. *J Int Med Res*. Jun 2021;49(6):3000605211028420. doi:10.1177/03000605211028420

130. Oakley I, Reece LP. Anesthetic implications for the patient with osteogenesis imperfecta. *AANA J*. Feb 2010;78(1):47-53.

131. Karabiyik L, Parpucu M, Kurtipek O. Total intravenous anaesthesia and the use of an intubating laryngeal mask in a patient with osteogenesis imperfecta. *Acta Anaesthesiol Scand*. May 2002;46(5):618-9. doi:10.1034/j.1399-6576.2002.460525.x

132. Arponen H, Bachour A, Bäck L, et al. Is sleep apnea underdiagnosed in adult patients with osteogenesis imperfecta? -a single-center cross-sectional study. *Orphanet J Rare Dis*. Dec 29 2018;13(1):231. doi:10.1186/s13023-018-0971-7

133. Lévy P, Kohler M, McNicholas WT, et al. Obstructive sleep apnoea syndrome. *Nat Rev Dis Primers*. Jun 25 2015;1:15015. doi:10.1038/nrdp.2015.15

134. Khan SI, Yonko EA, Carter EM, Dyer D, Sandhaus RA, Raggio CL. Cardiopulmonary Status in Adults with Osteogenesis Imperfecta: Intrinsic Lung Disease May Contribute More Than Scoliosis. *Clin Orthop Relat Res*. Dec 2020;478(12):2833-2843. doi:10.1097/corr.0000000000001400

135. Gross JB, Bachenberg KL, Benumof JL, et al. Practice guidelines for the perioperative management of patients with obstructive sleep apnea: a report by the American Society of Anesthesiologists Task Force on Perioperative Management of patients with obstructive sleep apnea. *Anesthesiology*. May 2006;104(5):1081-93; quiz 1117-8. doi:10.1097/00000542-200605000-00026

136. Practice guidelines for the perioperative management of patients with obstructive sleep apnea: an updated report by the American Society of Anesthesiologists Task Force on Perioperative Management of patients with obstructive sleep apnea. *Anesthesiology*. Feb 2014;120(2):268-86. doi:10.1097/aln.0000000000000053

137. Wallace MJ, Kruse RW, Shah SA. The Spine in Patients With Osteogenesis Imperfecta. *J Am Acad Orthop Surg*. Feb 2017;25(2):100-109. doi:10.5435/jaaos-d-15-00169

138. Gattrell WT, Logullo P, van Zuuren EJ, et al. ACCORD (ACcurate COnsensus Reporting Document): A reporting guideline for consensus methods in biomedicine developed via a modified Delphi. *PLoS Med*. Jan 2024;21(1):e1004326. doi:10.1371/journal.pmed.1004326
